# Redox-active molecules as organocatalysts for selective oxidative transformations – an unperceived organocatalysis field

**DOI:** 10.3762/bjoc.18.179

**Published:** 2022-12-09

**Authors:** Elena R Lopat’eva, Igor B Krylov, Dmitry A Lapshin, Alexander O Terent’ev

**Affiliations:** 1 N. D. Zelinsky Institute of Organic Chemistry, Russian Academy of Sciences, Leninsky Prospect 47, Moscow, 119991, Russia

**Keywords:** CH-functionalization, free radicals, hypervalent iodine, *N*-oxyl radicals, redox-active molecules

## Abstract

Organocatalysis is widely recognized as a key synthetic methodology in organic chemistry. It allows chemists to avoid the use of precious and (or) toxic metals by taking advantage of the catalytic activity of small and synthetically available molecules. Today, the term organocatalysis is mainly associated with redox-neutral asymmetric catalysis of C–C bond-forming processes, such as aldol reactions, Michael reactions, cycloaddition reactions, etc. Organophotoredox catalysis has emerged recently as another important catalysis type which has gained much attention and has been quite well-reviewed. At the same time, there are a significant number of other processes, especially oxidative, catalyzed by redox-active organic molecules in the ground state (without light excitation). Unfortunately, many of such processes are not associated in the literature with the organocatalysis field and thus many achievements are not fully consolidated and systematized. The present article is aimed at overviewing the current state-of-art and perspectives of oxidative organocatalysis by redox-active molecules with the emphasis on challenging chemo-, regio- and stereoselective CH-functionalization processes. The catalytic systems based on *N*-oxyl radicals, amines, thiols, oxaziridines, ketone/peroxide, quinones, and iodine(I/III) compounds are the most developed catalyst types which are covered here.

## Introduction

Organocatalysis can be defined as catalysis by small organic molecules, where an inorganic element is not a part of the active principle, that functions by donating or removing electrons or protons [1]. This definition distinguishes organocatalysis from the other two large catalysis areas: metal-complex catalysis and enzymatic/biocatalysis. The advantages of organocatalysts include their synthetic availability, wide opportunities for rational structure design, generally lower toxicity, air and water stability, metal-free nature, and the scalability of their production and use. These features have made organocatalysis a prospering area and an even more promising methodology for organic synthesis and technology [1–4]. The importance of organocatalysis was acknowledged recently by the awarding of the Nobel Prize in chemistry to Benjamin List and David MacMillan, the leading researchers in this field.

Selective oxidative transformations are crucially important for organic syntheses [5–9] and offer rich opportunities for the adaptation of organocatalysis. The diversity of such transformations spans from the functionalization of hydrocarbon raw materials to the fine organic synthesis, including functional group transformations, oxidative cyclizations, and oxidative coupling processes (also known as cross-dehydrogenative coupling), as well as late-stage CH-functionalization. Oxidation processes are recognized as a challenge in fine organic synthesis technology [10–11] due to selectivity problems and frequent need for toxic transition metal salts and stoichiometric oxidants. The development of organic chemistry is strongly affected by rising “green” demands to synthetic methodologies, including reduction of waste formation, low trace-metal content in drug compounds, high energy-efficiency and selectivity of processes. In the last decade researchers proposed numerous examples of redox-active organic molecules as effective organocatalysts meeting these requirements.

**Scheme 1:**
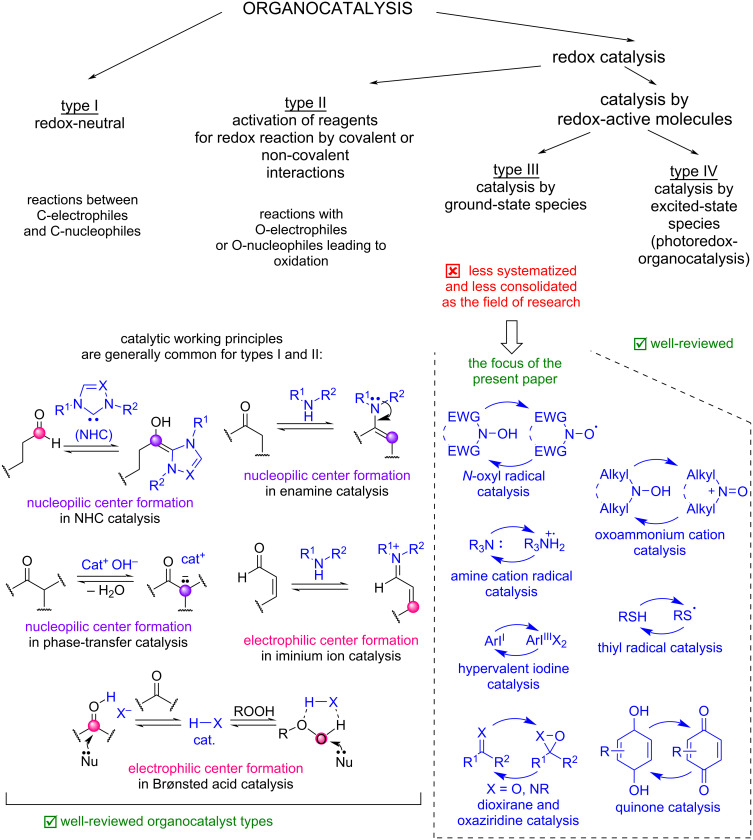
Organocatalysis classification used in the present perspective.

In spite of the large number of applications of organocatalysis in redox transformations, especially oxidative, the term “organocatalysis” is frequently perceived in the narrow sense centered around redox-neutral asymmetric organocatalysis, whereas organocatalysis by redox-active molecules stays in the shadows. For example, redox-active organic molecules are almost not mentioned in some recent overviews of compound types used in organocatalysis [3,12–13], except for photoredox catalysts [12–13]. The well-known and convenient classification of organocatalysts into Lewis bases, Lewis acids, Brønsted bases, and Brønsted acids [1] also leaves the redox-organocatalysts behind. Moreover, in numerous research papers employing redox-active molecules as catalysts the developed processes are not referred as “organocatalyzed”. In our opinion, the classification of redox-organocatalysis types can be useful for the consolidation of the research efforts in this field, which are currently “scattered” in the ocean of scientific literature. The classification clarifying the subject of the present perspective is shown in Scheme 1.

The organocatalyzed processes in which the oxidation states of atoms at reacting centers of substrates are not changed can be called “redox-neutral” (Scheme 1, type I). This is a very large group of reactions which may be the first coming to mind when talking about organocatalysis in general. Examples of typical reaction types for redox-neutral asymmetric organocatalysis are aldol reactions [13], Michael reactions [14–16], and Diels–Alder reactions [17–18].

The processes associated with changing oxidation states of atoms in substrates thus can be named “redox-organocatalyzed”. Within this section, two process types can be distinguished. In one an organocatalyst molecule is not reduced or oxidized itself but facilitates a reaction by activating the redox properties of reactants (Scheme 1, type II). In such processes the basic principles and organocatalyst structures are generally similar to those used in redox-neutral organocatalysis. These reactions are shortly discussed in the beginning of the main part of this perspective.

In the second type of redox-organocatalysis redox-active molecules are used, which undergo reduction and oxidation during the catalytic cycle turnover. The subclass of such reactions in which a catalyst is activated by light (photoredox catalysis, Scheme 1, type IV), especially visible, has gained much attention in the last decade. A considerable number of good reviews was published, both general [19–29] and specialized on the specific types of organophotoredox catalysts, such as quinone derivatives [30–31], carbon nitrides [32–33], eosin [34–36], 4CzIPN [37–38], Bodipy derivatives [39], methylene blue [40], pyrylium salts [41], and perylene diimides [42]. Photochemical processes involving enantioselective organocatalysis were also reviewed [28]. Taking into account the good coverage of organophotoredox-catalysis in these reviews we decided to focus on another, less systematized subclass of processes catalyzed by redox-active molecules in the ground state (Scheme 1, type III). There are a number of reviews on specific groups of redox-organocatalyzed oxidative transformations [43–56] and organocatalyzed enantioselective radical reactions were recently discussed [57]. However, the field remains not overviewed in general and it is not consolidated as a research field. The discussion below is organized according to the classes of redox-active molecules employed as organocatalysts. More specialized reviews are cited in the corresponding sections below. Of course, not all interesting processes catalyzed by redox-active molecules discovered in recent years are covered here. The present article is aimed at the summarization of main molecular structure types employed in redox organocatalysis, the consolidation of this research field, and highlighting possible areas of further development.

## Discussion

The present article is aimed at the demonstration of diversity and synthetic potential of redox-active molecules in the ground state as organocatalysts for selective oxidative transformations (Scheme 1, type III organocatalysis), as well as highlighting latest achievements in this field and perspective research trends. The discussed processes are classified according to the catalytically active species or key intermediates: *N*-oxyl radicals, oxoammonium cations, amine cation radicals, thiyl radicals, quinones, dioxiranes and oxaziridines, hypervalent iodine compounds, etc. However, some examples of organocatalyzed oxidative processes, in which an organocatalyst does not undergo oxidation or reduction but facilitates interaction between oxidant and substrate (Scheme 1, type II organocatalysis) are fluently discussed below to show the fundamental difference between type II and type III redox-organocatalysis.

### Organocatalysis by activation of redox properties of reagents

In this section we demonstrate examples of the main types of oxidative processes, in which an organocatalyst does not behave as a redox-active molecule itself but interacts with substrates and thus modulates their redox properties (Scheme 1, type II organocatalysis).

**Scheme 2:**
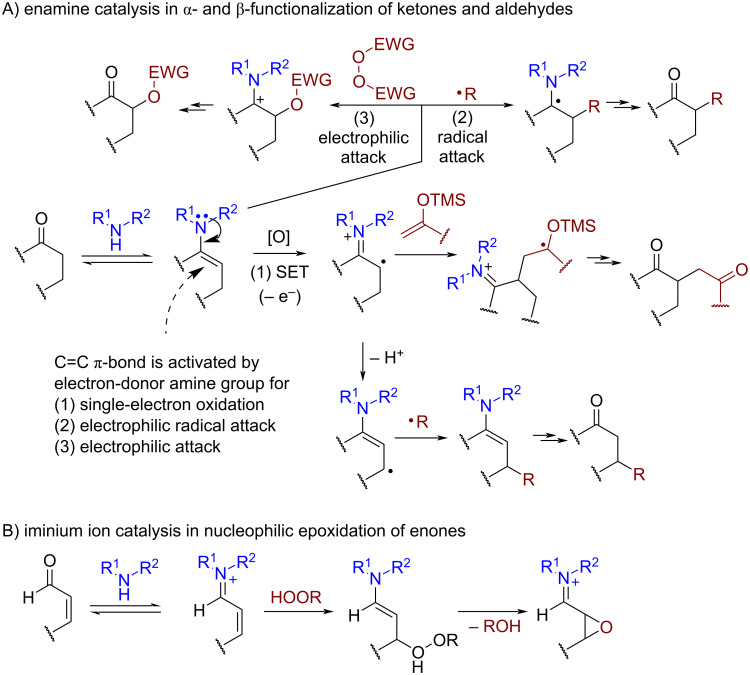
Oxidative processes catalyzed by amines.

Secondary amines play an important role in redox-neutral asymmetric organocatalysis by forming nucleophilic enamine intermediates or electrophilic iminium cations. The same principles are used in oxidative transformations, where an amine can play the role of chirality source and the activator of substrate for oxidation (Scheme 2).

In the enamine type of catalysis (Scheme 2A) the key enamine intermediate can undergo one-electron oxidation (route 1), electrophilic radical attack (route 2), or electrophile attack (route 3). The one-electron oxidation leads to the electrophilic cation radical which can further undergo addition to the electron-rich C=C bond [58] or proton loss followed by β-functionalization [59–61]. The iminium cation catalysis is used in the activation of electrophilic properties of enones for the nucleophilic epoxidation by hydroperoxides (Scheme 2B).

**Scheme 3:**
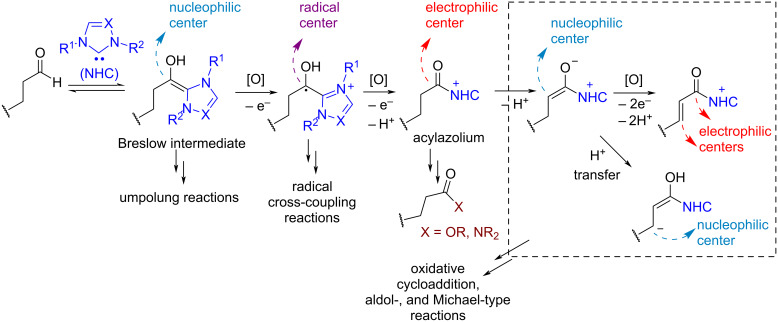
*N*-Heterocyclic carbene (NHC) catalysis in oxidative functionalization of aldehydes.

*N*-Heterocyclic carbene (NHC) organocatalysis [62] has found numerous applications in oxidative transformations, especially in the CH-functionalization of aldehydes (Scheme 3).

It is believed that NHCs reversibly form enaminols (Breslow intermediates, Scheme 3) from aldehydes [63]. In this transformation an electrophilic aldehyde carbon turns to a nucleophilic reacting center. In addition to umpolung reactions with electrophiles, Breslow intermediates can undergo oxidation with the formation of radical cations or electrophilic acylazolium cations. The acylazolium cation in turn can undergo nucleophilic attack resulting in C–O and C–N coupling, or deprotonation followed by the functionalization of α- and β-positions of the starting aldehyde. NHC-catalyzed photochemical processes [64] and oxidative cyclizations with heterocycle formation [65] were reviewed previously.

**Scheme 4:**
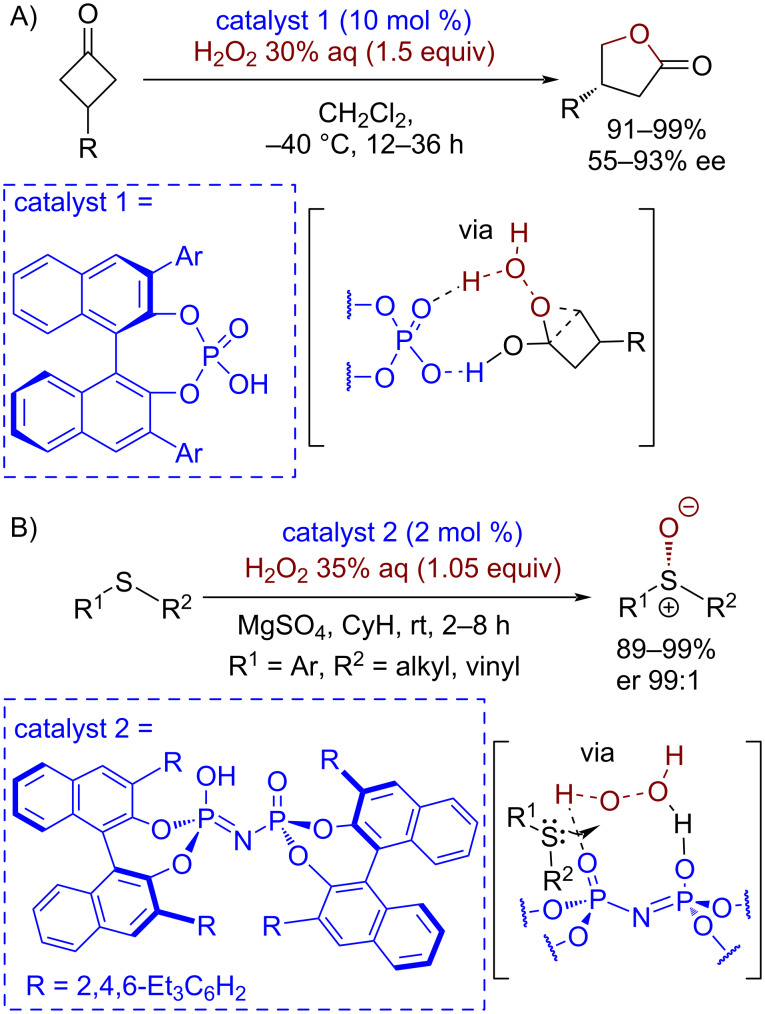
Examples of asymmetric oxidative processes catalyzed by chiral Brønsted acids.

Acidic molecules or hydrogen-bond donors are used as organocatalysts in oxidative processes for the activation of electrophilic properties of unsaturated substrates or for the activation of hydroperoxide oxidative properties. In Scheme 4A the proposed transition state for the Brønsted acid-catalyzed asymmetric Baeyer–Villiger reaction is shown, in which the organocatalyst forms hydrogen bonds with both H_2_O_2_ and cyclic ketones [66]. A chiral Brønsted acid was used as chirality source and activator of H_2_O_2_ for an asymmetric sulfoxidation reaction [67] (Scheme 4B). It is generally accepted that in asymmetric Brønsted acid catalysis the activation of both the electrophile and the nucleophile with the specific preorganization of the substrates by the catalyst is crucial for high enantioselectivity [67] (as in example A in Scheme 4). However, in example B the transition state without specific interactions between the sulfide and the catalyst is proposed. In this case, the acidic and basic sites of the catalyst are suggested to be involved in the activation of only hydrogen peroxide within a well-defined and deep chiral cavity. The enantioselective approach of sulfide to H_2_O_2_ is ensured by the sterically demanding structure of the catalyst.

It should also be noted that hydrogen bonding is considered as one of the key factors determining the selectivity of catalyst-free sulfoxidations [68]. In such reactions, the selectivity of sulfide oxidation by oxone (sulfoxide/sulfone ratio) was controlled by the solvent nature (deeper oxidation was observed in water than in ethanol). Brønsted acid catalysis by TsOH was also employed in a selective sulfoxidation employing PhI(OAc)_2_ as oxidant [69]. In this case another mode of catalysis was proposed, including the covalent bonding of the acid catalyst anion and the oxidant with the formation of PhI(OTs)OH as the catalytically active oxidative agent.

**Scheme 5:**
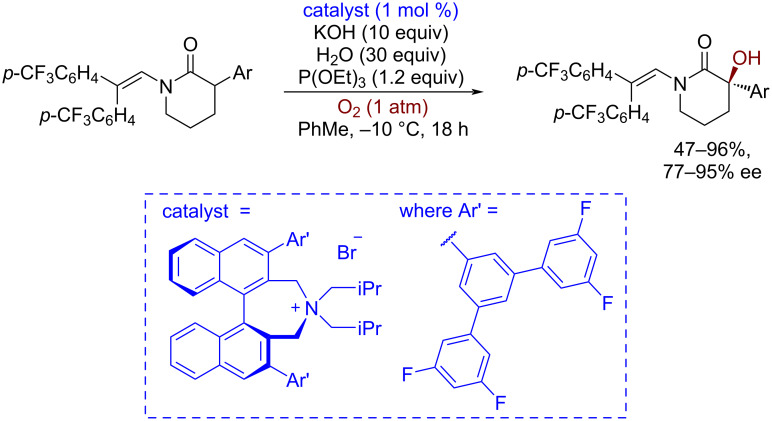
Asymmetric aerobic α-hydroxylation of lactams under phase-transfer organocatalysis conditions employing a chiral quaternary ammonium ion salt.

Asymmetric quaternary ammonium phase-transfer catalysts proved to be effective in the asymmetric nucleophilic epoxidation of electron-poor alkenes by hydroperoxides [70] and the asymmetric hydroxylation of enolizable carbonyl compounds employing O_2_ or H_2_O_2_ as terminal oxidants [71–72]. A recent achievement of the enantioselective hydroxylation of α‑aryl-δ-lactams by O_2_ is shown in Scheme 5 [73] as an example of such organocatalyzed reaction type. Triethyl phosphite is added to reduce a hydroperoxide, which is initially formed by the enolate oxidation with O_2_.

In summary, organocatalysis by the modulation of redox properties of reagents has much in common with redox-neutral organocatalysis. With the exception of free-radical processes, the distinguishing feature of these organocatalyzed oxidations frequently lies in the involvement of peroxides as O-nucleophiles or O-electrophiles instead of corresponding C-nucleophiles or C-electrophiles in redox-neutral organocatalyzed reactions.

### Organocatalysis by redox-active organic molecules

#### *N*-Oxyl radical catalysis

**Scheme 6:**
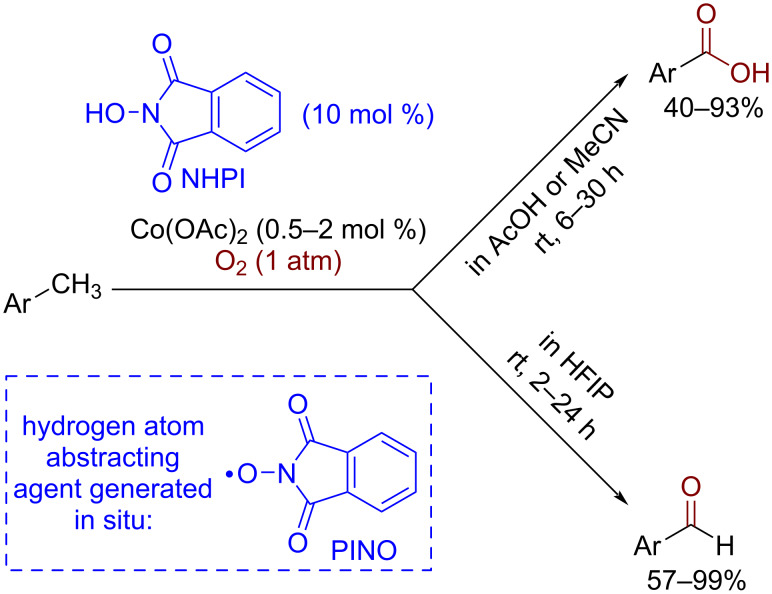
Selective CH-oxidation of methylarenes to aldehydes or carboxylic acids.

Reactive *N*-oxyl radicals generated in situ from the corresponding *N*-hydroxy compounds have found numerous applications in CH-functionalization reactions [74–77] due to their high reactivity in hydrogen atom abstraction and relatively slow self-decay. One of the most synthetically available and effective catalysts of this family is *N*-hydroxyphthalimide (NHPI). In the presence of co-catalysts, such as Co(OAc)_2_, it is partially oxidized by molecular oxygen to the phthalimide-*N*-oxyl radical (PINO) at room temperature and atmospheric oxygen pressure. The NHPI/Co(OAc)_2_ combination [78–80], also known as the Ishii catalytic system is one of the most effective in organic synthesis for the room temperature [78–79] aerobic oxidation of substrates with activated CH-bonds, such as alkylarenes (Scheme 6). At higher temperatures, even unactivated alkanes can be functionalized [75,81]. The NHPI/Co(OAc)_2_ system was successfully employed for the selective oxidation of methylarenes to aromatic carboxylic acids [78] (in AcOH medium) or aromatic aldehydes [79] (in 1,1,1,3,3,3-hexafluoropropan-2-ol, HFIP) at room temperature. The selectivity of aldehyde formation without the overoxidation to the carboxylic acid was explained by an inactivation of the aldehyde to further oxidation via the hydrogen bonding between the aldehyde and HFIP.

**Scheme 7:**
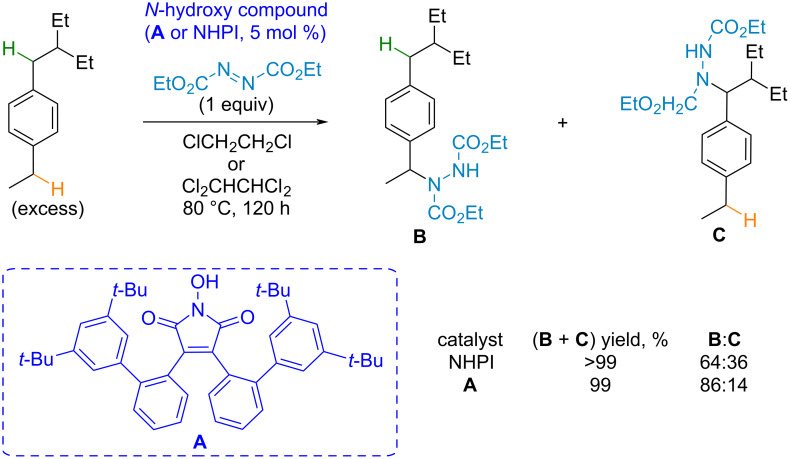
An example of the regioselective CH-amination by a sterically hindered imide-*N*-oxyl radical precursor.

The regioselective amination of benzylic positions in alkylarenes [82] and ethers [83] directed by steric effects was achieved by the development of sterically hindered “bowl-shaped” imide-*N*-oxyl radical precursors (Scheme 7). The presented example with a sterically hindered *N*-hydroxyimide catalyst shows the increase in selectivity compared to the reaction catalyzed by NHPI.

**Scheme 8:**
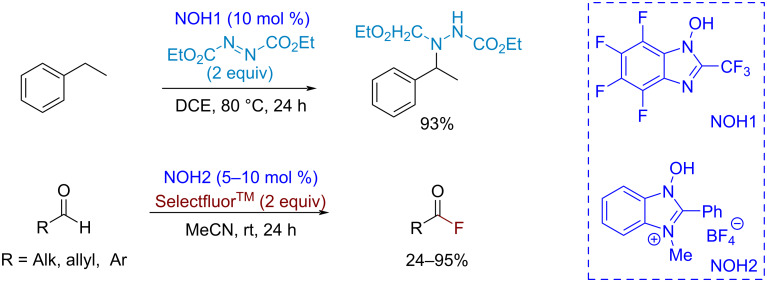
CH-amination of ethylbenzene and CH-fluorination of aldehydes catalyzed by *N*-hydroxybenzimidazoles, precursors of corresponding *N*-oxyl radicals.

In principle, the regioselectivity of a CH-functionalization can also be controlled by electronic effects of substituents in an *N*-oxyl radical, which influence the O–H bond-dissociation energy in the *N*-hydroxy compound and thus the hydrogen-abstracting activity of *N*-oxyl, respectively. Unfortunately, it is not easy to modify the structure of *N*-hydroxyimides. A step forward in the development of *N*-oxyl-catalyzed transformations was made with the introduction of a new catalyst type, *N*-hydroxybenzimidazoles [84]. The *N*-hydroxybenzimidazole core has several modification sites that allow one to control the properties of the catalyst over a wide range. The authors demonstrated the high efficiency of *N*-hydroxybenzimidazole catalysts in the benzylic CH-amination with diethyl azodicarboxylate and the CH-fluorination of aldehydes with Selectfluor^TM^ (Scheme 8).

**Scheme 9:**
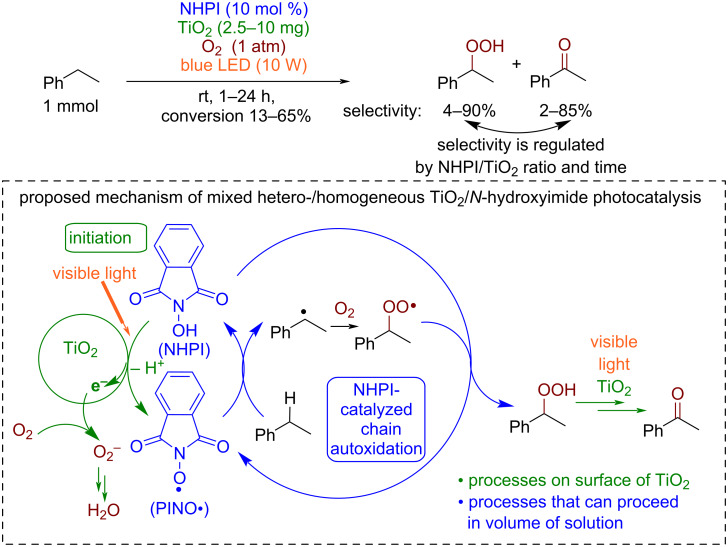
Mixed hetero-/homogeneous TiO_2_/*N*-hydroxyimide photocatalysis in the selective benzylic oxidation.

As was mentioned above, the *N*-hydroxyimide-organocatalyzed aerobic CH-oxidation at room temperature requires transition metal salts as co-catalysts. Transition metal salts are undesirable due to potential contamination of the target products or the inactivation of the catalytic system in the case of chelating substrates [80]. In order to avoid the usage of transition metal salts, metal-free oxidative systems for the generation of imide-*N*-oxyl radicals were proposed. In the majority of such systems, an *N*-hydroxyimide undergoes single-electron oxidation photochemically by a photoredox-catalyst or electrochemically on an anode. An example of the photochemical aerobic benzylic CH-oxidation employing a heterogeneous photoredox catalyst, nanosized TiO_2_, was demonstrated by our group [85] (Scheme 9).

Mixing of NHPI and TiO_2_ leads to the emergence of visible light absorbance, apparently, due to the interaction of NHPI with the TiO_2_ surface. It is noteworthy that NHPI or TiO_2_ alone demonstrated negligible activity compared to their simultaneous use. Upon the irradiation by blue LED, PINO radicals are formed on the TiO_2_ surface and diffuse in the volume of solution, where the radical-chain PINO/NHPI-catalyzed autoxidation proceeds with the selective formation of a benzylic hydroperoxide (Scheme 9), a product that frequently decomposes in the presence of transition metal ions or photoredox catalysts. It was shown that the peroxide is converted to the ketone on the TiO_2_ surface under visible light irradiation. The advantages of this mixed hetero-/homogeneous catalytic system include the easy separation of the products from NHPI and TiO_2_, recyclability, selectivity control by TiO_2_/NHPI ratio, and high efficiency at low TiO_2_ loading due to the radical chain nature of the homogeneous process, which once initiated on the TiO_2_ surface, can produce multiple molecules of product without additional light absorption.

**Scheme 10:**
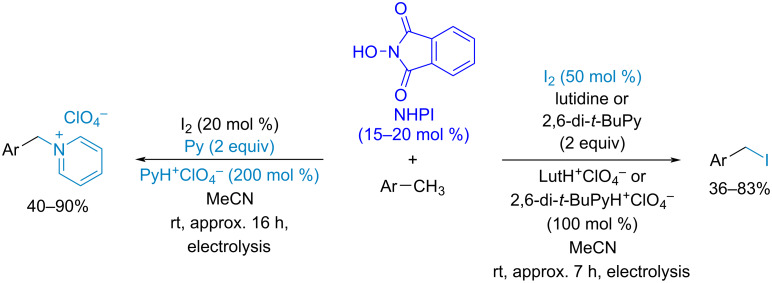
Electrochemical benzylic iodination and benzylation of pyridine by benzyl iodides generated in situ. Electrolysis conditions: Constant current electrolysis until anodic potential 1.12 V vs Fc/Fc^+^, divided cell, reticulated vitreous carbon (RVC) anode(+).

Another approach to the generation of imide-*N*-oxyl radicals under mild conditions without transition metal salts as co-catalysts employs electric current as the oxidant [74,76,80,86–88]. To facilitate the anodic oxidation of *N*-hydroxyphthalimide, basic pyridine derivatives are used as the *N*-hydroxyphthalimide proton acceptors [87]. In many cases electrolysis can be performed in the galvanostatic mode in a simple undivided cell, which is convenient for multigram-scale syntheses [86]. The selective allylic [86] and benzylic [80] CH-oxidation to the corresponding carbonyl compounds was achieved. Compared to the direct anodic oxidation of organic substrates, the *N*-oxyl-mediated indirect electrolysis proceeds at lower potentials, demonstrates wider application scope, robustness, and selectivity [74]. Recently, an electrochemical NHPI/PINO-mediated benzylic iodination was achieved using lutidine or 2,6-di*-tert*-butylpyridine as bases with low nucleophilicity [89] (Scheme 10). When pyridine was used instead 2,6-disubstituted pyridines its *N*-benzylation by benzyl iodides was observed.

**Scheme 11:**
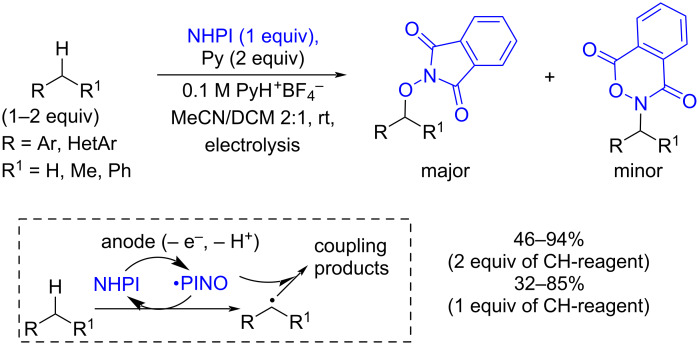
Electrochemical oxidative C–O/C–N coupling of alkylarenes with NHPI. Electrolysis conditions: Constant current electrolysis (2–3 F/mol) until potential rised by 0.5–0.8 V above initial potential; undivided cell, reticulated vitreous carbon (RVC) anode(+):Pt cathode(−).

Besides classical NHPI/PINO-catalyzed CH-functionalization processes, there is a significant number of works in which PINO plays the role of both the catalyst for C–H bond cleavage and the reagent intercepting the resultant C-centered radical [90]. As a rule, stoichiometric amounts of strong oxidants ((NH_4_)_2_Ce(NO_3_)_6_ [91], PhI(OAc)_2_ [92], *t*-BuOO*t*-Bu [93], etc.) are needed to generate a sufficient concentration of PINO radicals for the effective trapping of C-centered radicals and excess amounts of a CH-reagent are necessary for high selectivity. The electrochemical oxidative coupling of alkylarenes with NHPI was realized recently [94] (Scheme 11). In this process the formation of benzylic radicals is catalyzed by PINO radicals. According to the mechanism supported by quantum chemical calculations, the mixture of C–O and C–N products is formed as a result of the attack of the benzyl radical on the O or N atom of the PINO radical, respectively. It should be noted that this method works well even without the excess of CH-reagent [94].

The formation of the mixture of C–O and C–N coupling products may be the main factor limiting the application of this method. The selective C–O coupling can be potentially achieved in the future by the development of novel *N*-oxyl radicals without the *N*-reactivity in coupling. General problems to be solved in *N*-oxyl radical catalysis are their gradual decay under the reaction conditions, which restrains catalyst recycling and substrate scope, and the limited arsenal of *N*-oxyl radicals with varying hydrogen-abstracting activity for the functionalization of various substrates and the achievement of high chemo- and regioselectivity. The most synthetically available and widely used *N*-hydroxyimide catalysts are inclined to the nucleophilic attack on carbonyl groups resulting in catalyst deactivation. For example, NHPI catalysis is not compatible with primary and secondary amines [75]. To sum up, the future development of *N*-oxyl radical catalysis is expected to be associated with discoveries of new structural types of *N*-oxyl radicals with improved stability and tunable reactivity by structure modification.

#### Amine-*N*-oxyl radical/oxoammonium cation catalysis

Compared to the *N*-oxyl radical catalysis discussed above, where reactive *N*-oxyl radicals represent an active organocatalyst form generated in situ, in oxoammonium cation catalysis [74,76,78,95] less reactive and less electron-deficient *N*-oxyl radicals (usually isolable and storable amine-*N*-oxyl radicals) are used as precursors of the active oxidants, oxoammonium cations.

**Scheme 12:**
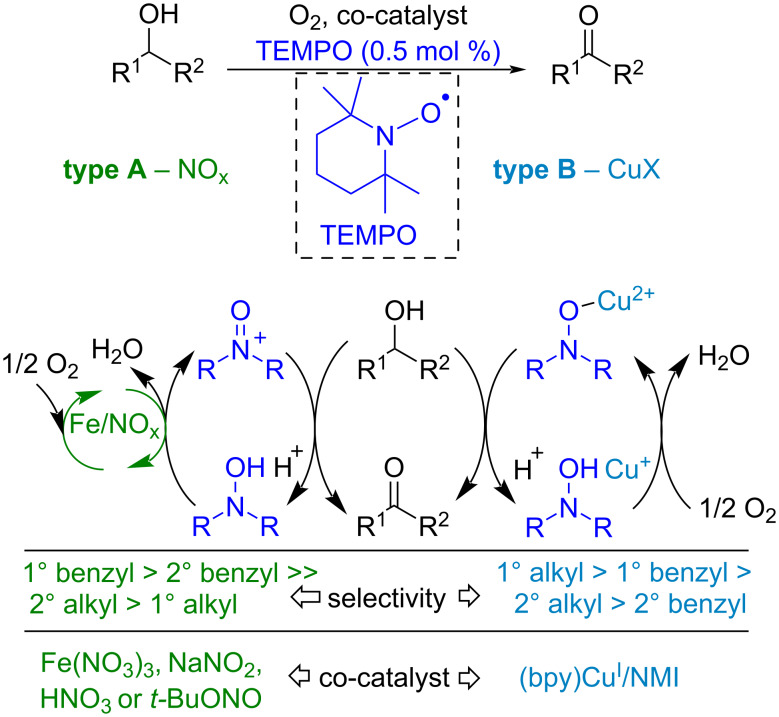
Chemoselective alcohol oxidation catalyzed by TEMPO.

This oxidative organocatalysis type is highly important for the chemoselective oxidation of alcohols. Two fundamentally different mechanisms have been proposed for the oxidation of alcohols [96] (Scheme 12). When using transition metals such as Cu(I) as co-catalyst, both aminoxyl radicals and metal ions serve as one-electron oxidants in a joint two-electron oxidation. In this system, primary aliphatic alcohols can be selectively oxidized in the presence of secondary alcohols. In the case of co-catalysts Fe(NO_3_)_3_ or NO_x_ species (NaNO_2_, HNO_3_, *t-*BuONO), an aminoxyl is oxidized in situ to an oxoammonium cation, which oxidizes alcohols. Fe and NO_x_-based methods demonstrate lower functional group compatibility and different chemoselectivity: secondary and benzyl alcohols are more easily oxidized.

The great diversity of catalytic systems based on amine-*N*-oxyl radicals for alcohol oxidation was proposed [74,76–77,95]. Amine-*N*-oxyl organocatalysts with enhanced catalytic activity were developed by the modification of the hydrocarbon cyclic structure making the NO reactive center more sterically available [97] or by the introduction of electron-withdrawing groups (electronically tuned nitroxyl radical catalysis) [98–100]. More recently, the application of chiral electronically tuned nitroxyl radicals for the kinetic resolution of racemic alcohols [99] and for the oxidation of benzylic cyclic ethers to lactones [100] was demonstrated.

**Scheme 13:**
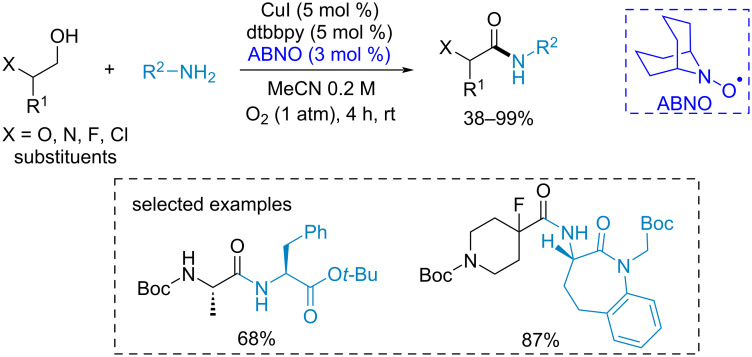
ABNO-catalyzed oxidative C–N coupling of primary alcohols with primary amines.

The CuI/9-azabicyclo[3.3.1]nonane *N*-oxyl (ABNO) catalytic system successfully promotes the oxidative coupling of alcohols with primary amines [101] (Scheme 13). The reaction proceeds with the preservation of stereocenters. It is tolerant to a wide range of functional groups, which makes it compatible with natural substances and complex biologically active compounds.

**Scheme 14:**
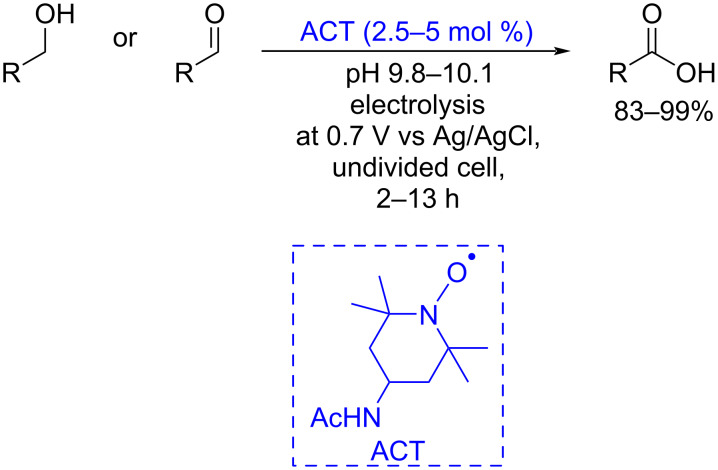
ACT-catalyzed electrochemical oxidation of primary alcohols and aldehydes to carboxylic acids.

TEMPO and its derivatives are successfully used as electrocatalysts. 4-Acetamido-2,2,6,6-tetramethylpiperidin-1-oxyl (ACT) allows for the oxidation of alcohols and aldehydes to carboxylic acids by controlled potential electrolysis, while maintaining the stereocenter configuration in the R-substituent [102] (Scheme 14). The method is also suitable for molecules with chelating pyridine moieties.

**Scheme 15:**
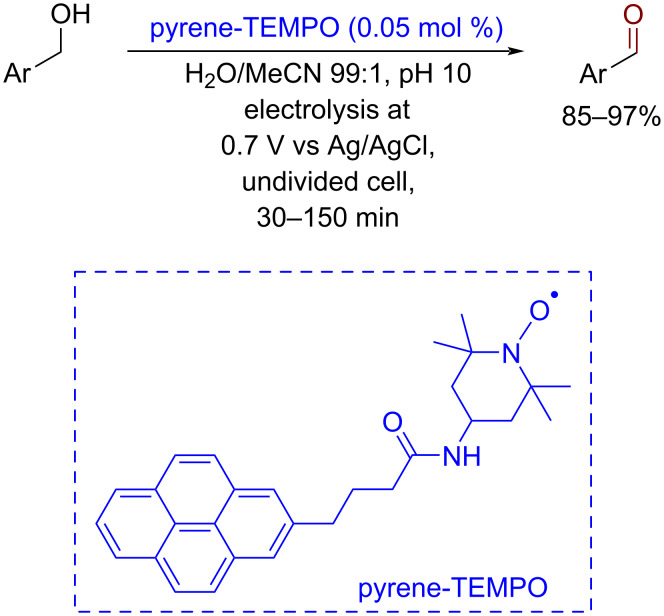
Electrocatalytic oxidation of benzylic alcohols by a TEMPO derivative immobilized on a graphite anode by π–π stacking interactions.

An outstanding electrocatalytic efficiency (TON up to 2000) was achieved using the TEMPO derivative non-covalently immobilized on the surface of a carbon cloth anode due to the π–π stacking interaction between the pyrene fragment of the catalyst and the electrode surface [103] (Scheme 15). However, this method is not compatible with amino and nitro groups due to side processes on the electrodes.

**Scheme 16:**
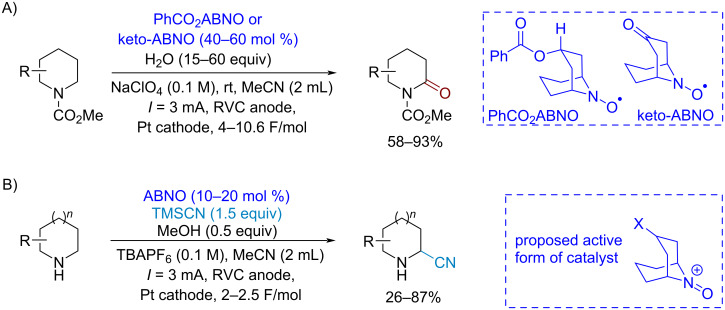
Electrochemical oxidation of carbamates of cyclic amines to lactams and oxidative cyanation of amines catalyzed by ABNO-type amine-*N*-oxyl radicals.

Cyclic carbamates were oxidized to lactams using ABNO derivatives as electrocatalysts [104] (Scheme 16A). The substrates with easily oxidizable pyrazole and oxazole fragments reacted successfully under these conditions. Under similar conditions the oxidative ABNO-catalyzed α-cyanation of amines was realized with no need for N-protecting groups [105] (Scheme 16B). The key reactive species proposed in these electrochemical reactions are the oxoammonium cations formed from the amine-*N*-oxyl catalyst at the anode. The oxoammonium cation promotes the formal hydride transfer from the substrate, resulting in the formation of the hydroxylamine and substrate-derived iminium cation, which undergoes nucleophilic addition.

#### Amine cation radical catalysis

**Scheme 17:**
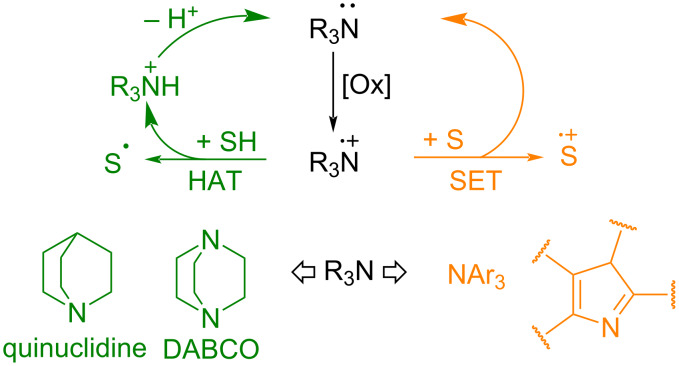
Hydrogen atom transfer (HAT) and single-electron transfer (SET) as basic principles of amine cation radical oxidative organocatalysis.

The single-electron oxidation of amines leads to amine cation radicals. Amine cation radicals which are stable to self-decay are used in oxidative organocatalysis as hydrogen atom acceptors or one-electron oxidants (Scheme 17). The stability of cation radical to self-decay is achieved in bicyclic structures where the cleavage of a hydrogen atom from the carbon atom next to nitrogen is unfavorable (DABCO, quinuclidine) and in the case of triarylamines containing no hydrogen atoms at carbon atoms connected to the cation radical center. Cation radicals of aromatic amines or *N*-heterocyclic cation radicals are usually involved in electron transfer processes due to their low affinity to hydrogen atoms (instability of the corresponding ammonium cations), whereas cation radicals of aliphatic bicyclic amines are effective in hydrogen atom abstraction (Scheme 17).

**Scheme 18:**
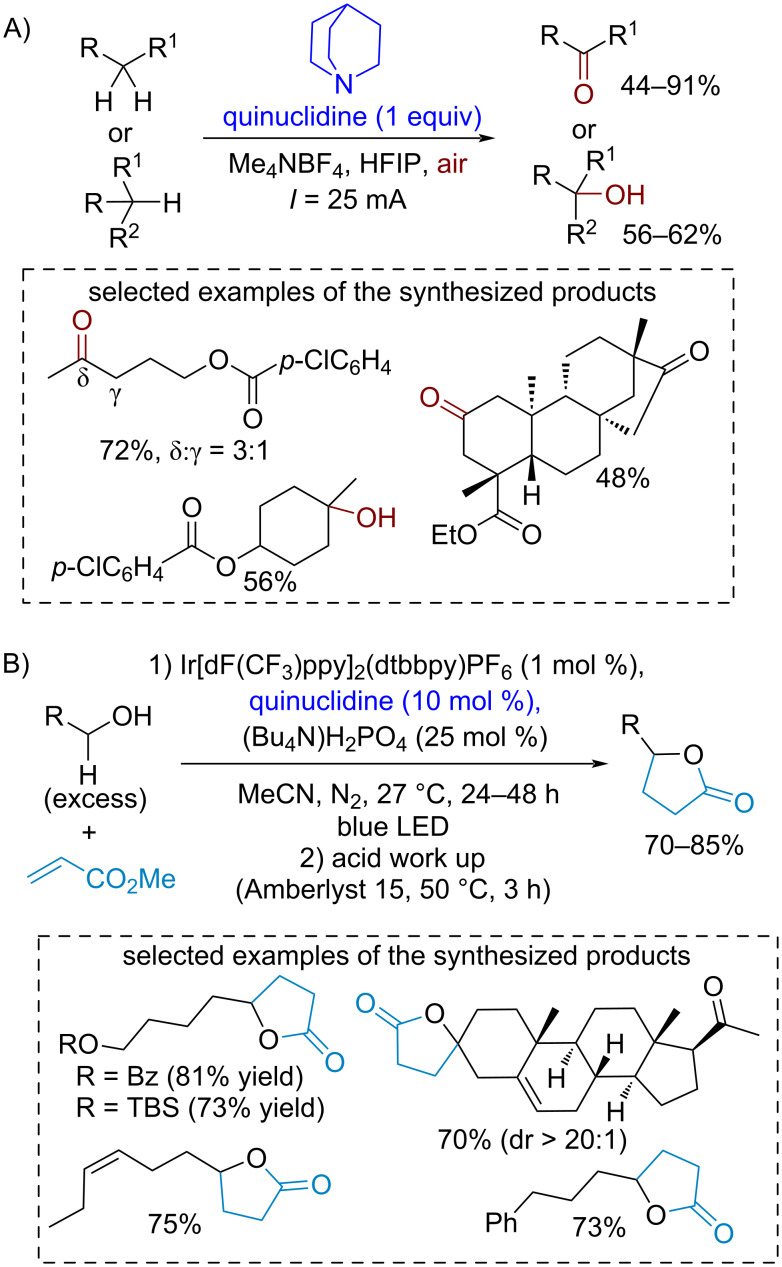
Electrochemical quinuclidine-catalyzed oxidation involving unactivated C–H bonds.

For example, the electrochemical CH-oxidation of unactivated substrates mediated by quinuclidine was demonstrated [106] (Scheme 18A). In several cases, a good regioselectivity was achieved for complex molecules. Quinuclidine cation radicals were also involved in the generation of nucleophilic α-hydroxyalkyl radicals from alcohols for the addition to the electron-deficient C=C bond of methyl acrylate followed by lactonization [107] (Scheme 18B). An interesting idea realized in this process is the alcohol activation for α-hydrogen atom abstraction by hydrogen bonding between the alcohol OH group and dihydrophosphate anions. It should be noted that alcohol-derived α-hydroxy radicals frequently do not undergo successful C–C coupling due to the side process of their overoxidation to aldehydes or ketones. In another work, 3-acetoxyquinuclidine showed improved yields compared to unsubstituted quinuclidine in the α-functionalization of *N*-Boc-substituted secondary amines [108], showcasing an amine cation radical activity tuning by the introduction of an electron-withdrawing acetoxy group.

**Scheme 19:**
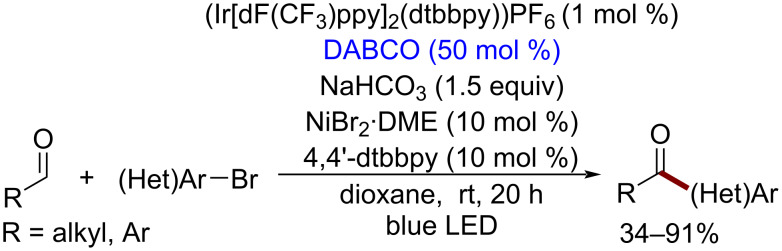
DABCO-mediated photocatalytic C–C cross-coupling involving aldehyde C–H bond cleavage.

The DABCO cation radical is less reactive compared to quinuclidine-derived cation radicals. It was involved in the Ni-catalyzed oxidative C–C cross-coupling involving aldehyde C–H bond cleavage with the formation of acyl radicals according to the proposed mechanism [109] (Scheme 19).

**Scheme 20:**
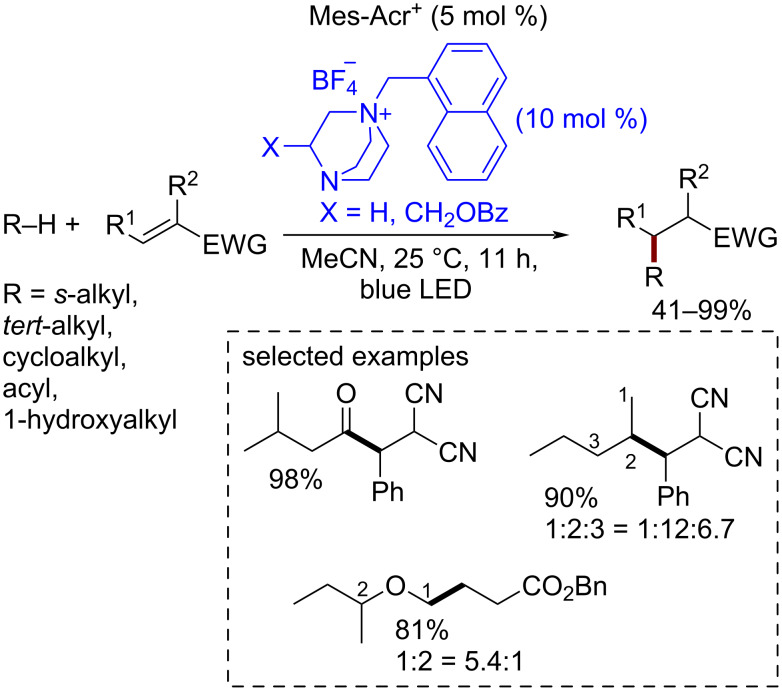
DABCO-derived cationic catalysts in inactivated C–H bond cleavage for alkyl radical addition to electron-deficient alkenes under photoredox catalysis conditions.

A novel family of redox-organocatalysts based on the synthetically available DABCO fragment was proposed [110] (Scheme 20). The quaternization of one of the nitrogen atoms of DABCO leads to a dramatically improved hydrogen atom abstracting activity which allowed the authors to perform the CH-functionalization of alkanes by electron-deficient alkenes. Photoredox catalysis was employed for the one-electron oxidation of the DABCO-derived organocatalyst to the dication radical. The authors noted that the introduction of a substituent X near the radical center further improves the site-selectivity of CH-functionalization. Important advantages of this organocatalyst family are synthetic availability and activity tuning by variation of substituents.

**Scheme 21:**
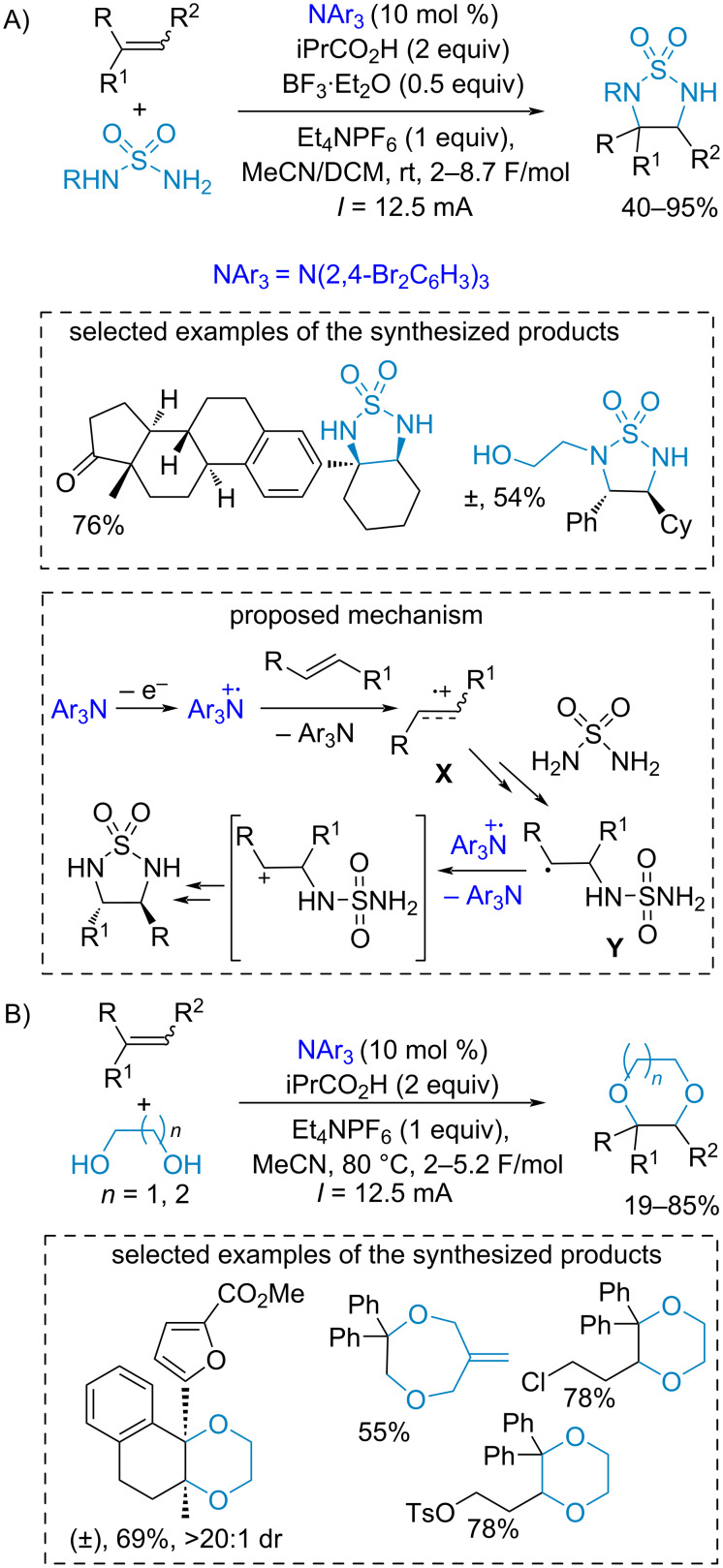
Electrochemical diamination and dioxygenation of vinylarenes catalyzed by triarylamines.

In contrast to the aliphatic amine cation radicals which typically abstract H-atoms, the cation radicals of aromatic amines are used mainly as mediators of oxidative electron transfer (SET mechanism). For example, one-electron oxidative properties of triarylamine cation radicals were used for the vinylarene difunctionalization with the formation of thiadiazolidine 1,1-dioxides that can be further transformed to the corresponding 1,2-diamines [111] (Scheme 21A). The proposed reaction mechanism suggests the generation of a triarylamine radical cation, which oxidizes the vinylarene by a SET mechanism. The resultant vinylarene cation radical **X** is attacked by the sulfamide nucleophile with **Y** formation. The second oxidative SET leads to the cyclization affording the final product. Similarly, the dioxygenation of vinylarenes by diols was realized affording 1,4-dioxaheterocycles [112] (Scheme 21B).

**Scheme 22:**
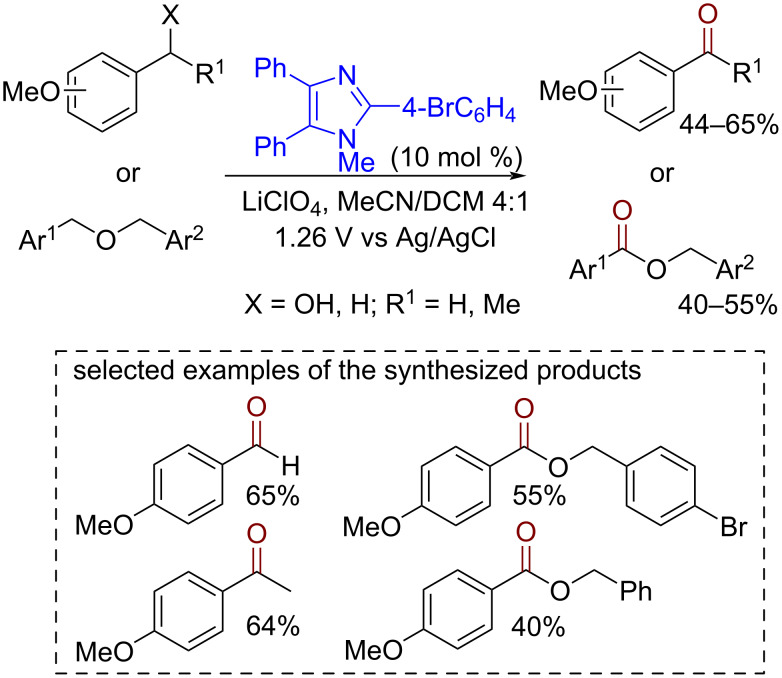
Electrochemical benzylic oxidation mediated by triarylimidazoles.

Triarylimidazoles have found similar applications as mediators for electrochemical oxidations [113] (Scheme 22). The reaction was suggested to proceed via a SET mechanism. The first electron transfer occurs from the aromatic system to the organocatalyst cation radical. After the deprotonation of the benzylic position a benzylic radical is formed. Then, a benzylic cation is produced by a second oxidative electron transfer followed by nucleophile addition (if X = H) or proton cleavage in the case of X = OH.

Amine mediators are also suitable for the generation of radical species from CH-acidic substrates, such as β-dicarbonyl compounds. This reactivity was used in the dehydrogenative annulation of *N*-allyl amides with β-dicarbonyl compounds with the formation of N-heterocycles [114].

#### Thiyl radical catalysis

Thiyl radicals [115] can undergo hydrogen atom abstraction from substrates and reversible addition to double C–C bonds [115–117]. The corresponding thiols can play the role of hydrogen atom donors. The fast hydrogen atom abstraction from thiols by nucleophilic radicals with the formation of electrophilic thiyl radicals ensured the wide application of thiols in so-called polarity reversal catalysis [118–121].

**Scheme 23:**
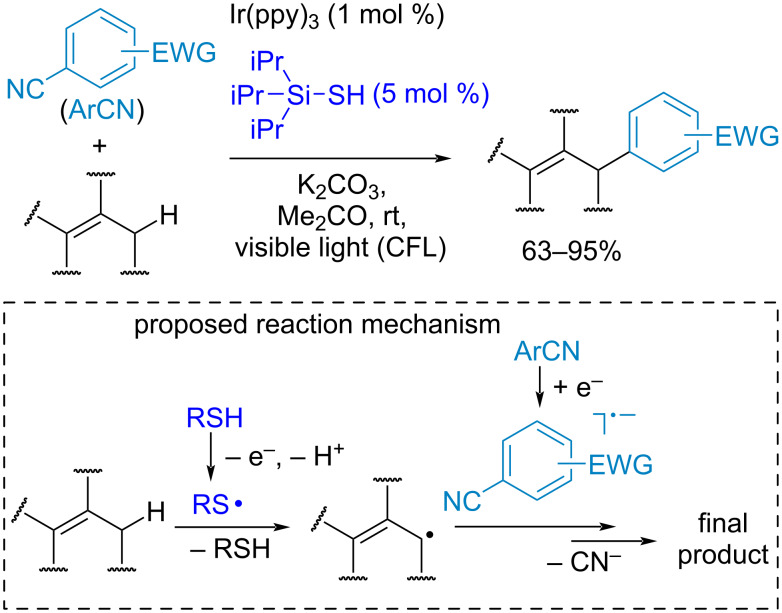
Thiyl radical-catalyzed CH-arylation of allylic substrates by aryl cyanides.

In a number of works thiyl radicals were used for hydrogen atom abstraction in the arylation of activated CH-reagents (for example, amines, alkenes, benzyl ethers) by electron-deficient aryl cyanides under photoredox conditions. An example of such process is presented in Scheme 23 [122]. The key stages of the proposed mechanism include the photoredox-catalyzed generation of a thiyl radical and anion radical from ArCN, and the allylic hydrogen atom abstraction by the thiyl radical. The final product is formed by the coupling of the ArCN anion radical with an allylic radical.

**Scheme 24:**
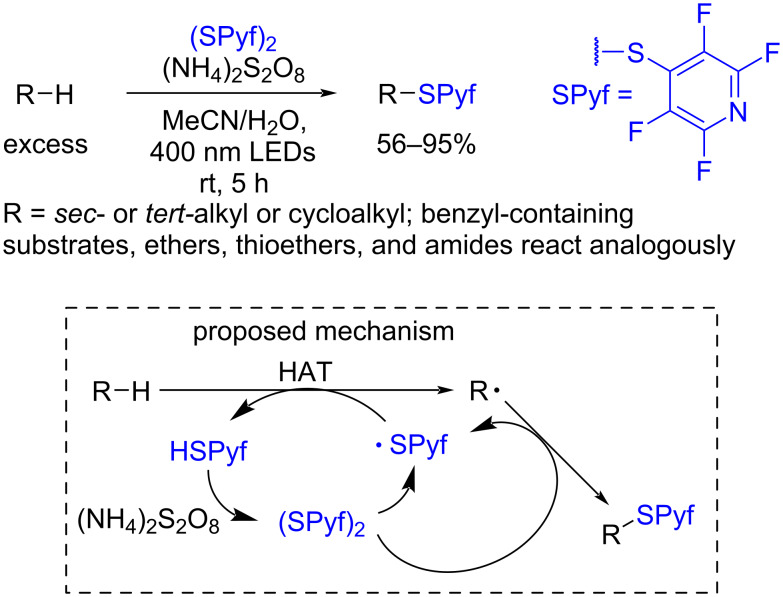
Synthesis of redox-active alkyl tetrafluoropyridinyl sulfides by unactivated C–H bond cleavage by tetrafluoropyridinyl thiyl radicals (SPyf).

As was mentioned above, thiyl radicals were mainly applied for the cleavage of activated C–H bonds. Recently, the tetrafluoropyridinyl thiyl radical (SPyf, Scheme 24) was proposed as a hydrogen atom abstracting agent for unactivated C–H bonds of alkanes and other CH-reagents [123] (Scheme 24). It was generated by the irradiation of the corresponding disulfide with 400 nm LEDs. In the proposed catalytic cycle tetrafluoropyridinyl thiol HSPyf is reoxidized to the disulfide by (NH_4_)_2_S_2_O_8_. The disulfide (SPyf)_2_ is also consumed in the thiolation of C-centered radicals affording the SPyf radical and target sulfides, which are in turn useful C-centered radical precursors for photoredox-catalyzed reactions [123].

The high efficiency of the thiolation process was explained by the unusual feature in the reactivity of the HSPyf/(SPyf)_2_ pair compared to other thiols and disulfides revealed by DFT calculations. In the case of the SPyf moiety the free-energy barrier for HAT between C-centered radicals and HSPyf is higher than the barrier for an SPyf group transfer between the C-centered radical and (SPyf)_2_, whereas for Me-, CF_3_-, Ph-, and C_6_F_5_-derived thiols and disulfides HAT is more favorable than thiyl group transfer [123].

#### Quinone catalysis

**Scheme 25:**
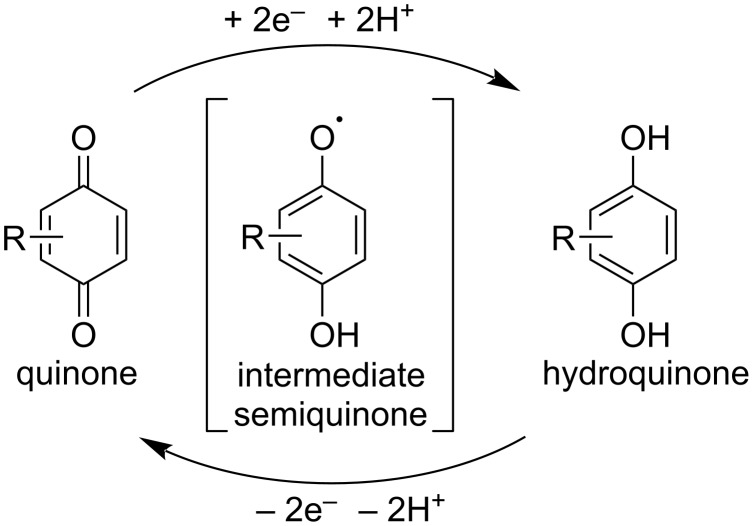
Main intermediates in quinone oxidative organocatalysis.

Quinones are well known as redox-active cofactors in biochemical processes and have found wide synthetic application as redox-catalysts [124–125] or photoredox catalysts [30–31] for selective oxidations and also as stoichiometric oxidants [126]. Electron-withdrawing groups are used to increase oxidative properties, the most known examples are 2,3-dichloro-5,6-dicyano-1,4-benzoquinone (DDQ) [126] and 2,3,5,6-tetrachloro-1,4-benzoquinone (*p*-chloranil). In a typical catalytic cycle, the quinone molecule performs two-electron oxidation to form the hydroquinone, which is then reoxidized by terminal oxidants (Scheme 25). However, radical semiquinone intermediates can also be formed and participate in the oxidation of substrates.

**Scheme 26:**
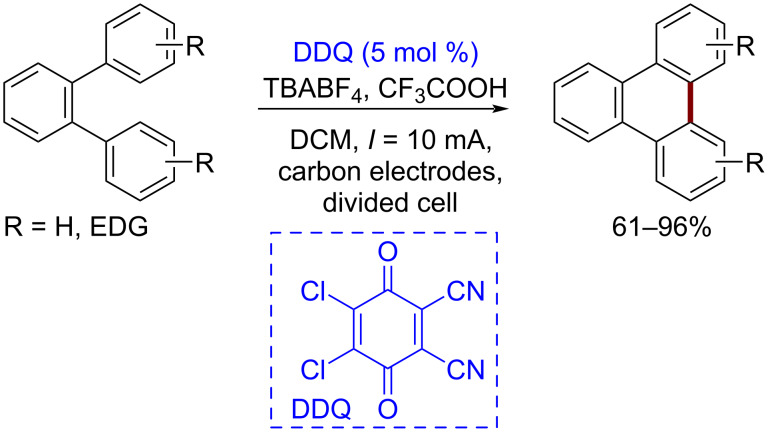
Electrochemical DDQ-catalyzed intramolecular dehydrogenative aryl–aryl coupling.

DDQ is a popular mediator for oxidation reactions. It has been used for intramolecular dehydrogenative C–C bond formation between aromatic groups [127]. Using this method, the formation of polyaromatic systems was achieved in good yields (Scheme 26).

**Scheme 27:**
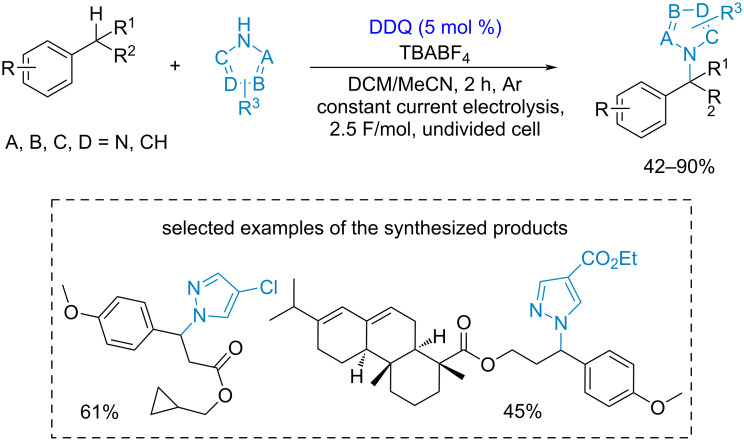
DDQ-mediated cross-dehydrogenative C–N coupling of benzylic substrates with azoles.

The cross-dehydrogenative C–N coupling of benzylic substrates with azoles was developed [128] (Scheme 27). In the proposed mechanism DDQ participated in benzylic C–H bond cleavage. The C–N bond of the final product is formed as a result of the nucleophilic attack of azole on a benzylic cation. A two-fold molar excess of azoles was used.

**Scheme 28:**
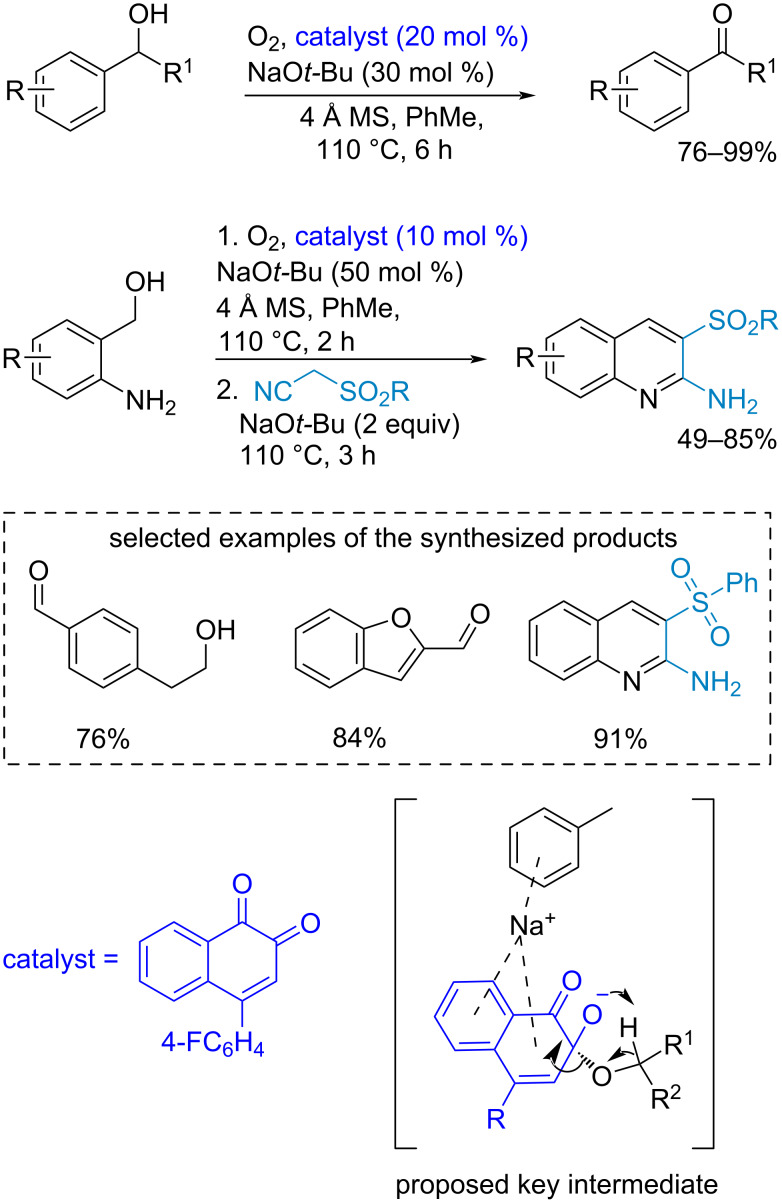
Biomimetic *o*-quinone-catalyzed benzylic alcohol oxidation.

A biomimetic oxidation of benzyl alcohols was developed using *o*-naphthoquinone [129] (Scheme 28). The reaction shows regioselectivity toward benzylic alcohol oxidation. This process could be included in one-pot synthesis strategies. The key step of the reaction is a 1,5-hydrogen transfer (the suggested intermediate is depicted in Scheme 28).

**Scheme 29:**
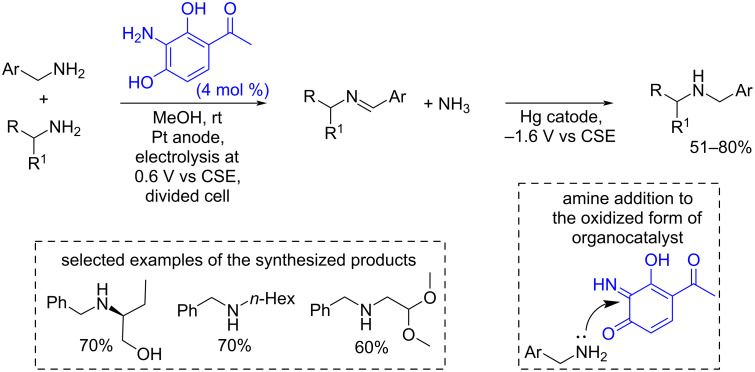
Electrochemical synthesis of secondary amines by oxidative coupling of primary amines and benzylic amines.

Quinone derivatives could also be generated in situ by anodic oxidation of phenolic compounds. An example of such process is the electrocatalytic biomimetic synthesis of secondary amines by *o*-azaquinone catalysis [130] (Scheme 29). Under anodic oxidation conditions imine derivatives were formed, which were reduced to amines in the second step (one pot) on a mercury cathode. The mercury cathode can be replaced by a carbon cathode, however, the yields are lower in this case [130].

#### Dioxirane and oxaziridine catalysis

**Scheme 30:**
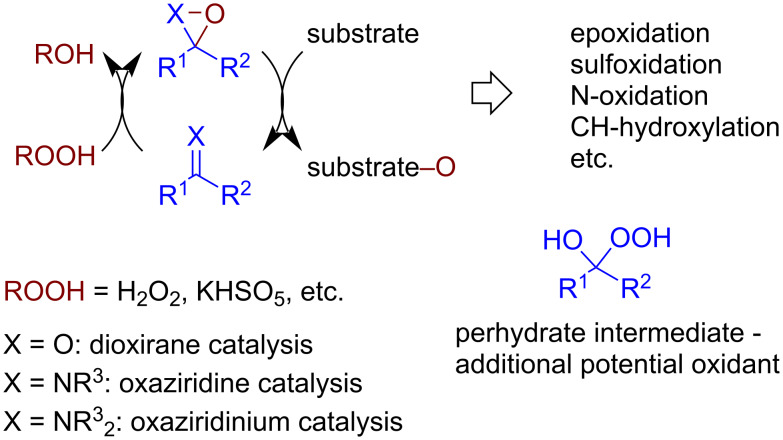
General scheme of dioxirane and oxaziridine oxidative organocatalysis.

In dioxirane or oxaziridine catalysis, the nucleophilic attack of a hydroperoxide (terminal oxidant) on a ketone or imine, respectively, is followed by intramolecular cyclization with O–O bond cleavage and the formation of a strained 3-membered ring, an electrophilic oxygen atom donor for oxidative processes (Scheme 30). It should be noted that in the ketone–H_2_O_2_ system the adducts of H_2_O_2_ and ketones (perhydrates) can also be active oxidative species [51].

**Scheme 31:**
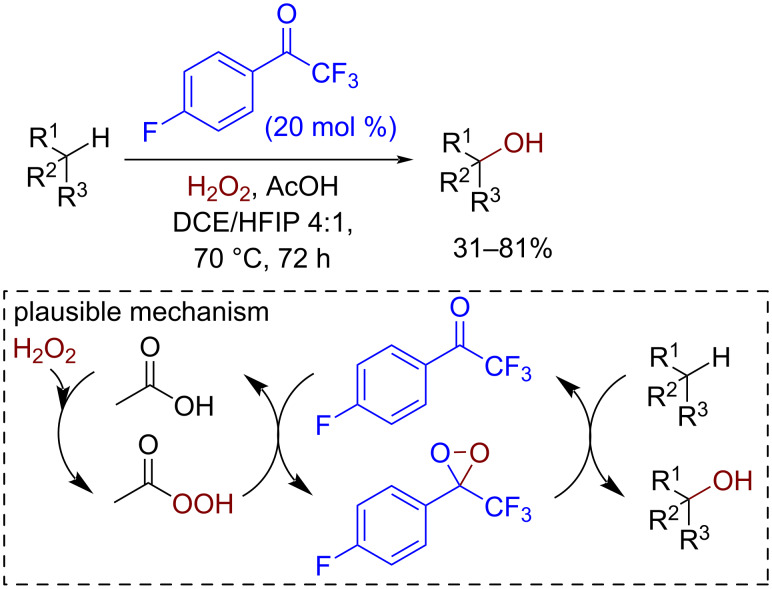
Dioxirane organocatalyzed CH-hydroxylation involving aliphatic C(sp^3^)–H bonds.

Dioxiranes formed from ketones and hydroperoxides are electrophilic oxygen transferring agents used in epoxidation (including asymmetric variants) [131–132], CH-hydroxylation, and other oxidation processes [132–134]. Fluorinated substituents in the ketone molecule are used to achieve higher electrophilicity and reactivity. Both radical and ionic mechanisms were reported for CH-functionalization reactions [134–135]. An example of an oxidative hydroxylation involving aliphatic C(sp^3^)–H bonds is presented in Scheme 31 [136].

**Scheme 32:**
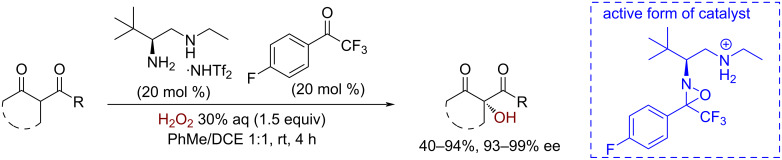
Enantioselective hydroxylation of CH-acids catalyzed by chiral oxaziridines.

An enantioselective oxidation can be achieved by combining a ketone catalyst with a chiral amine [137]. The resulting chiral oxaziridine intermediate promotes the hydroxylation of CH-acidic 1,3-dicarbonyl compounds in high yields and enantioselectivities (up to 99% ee) (Scheme 32). It should be noted that other α-carbonyl CH bonds are not affected under these reaction conditions, which makes the method suitable for the late-stage functionalization of complex molecules.

The method for selective remote CH-hydroxylation involving unactivated C(sp^3^)–H bonds employing oxaziridinium organocatalysis was developed recently [138]. The advantage of this method is the compatibility with secondary alcohol groups, which are not oxidized and can be used as a directing moiety. The use of HFIP as a strong hydrogen bond donor solvent protecting alcohols from oxidation was considered as the key factor for the high chemoselectivity.

#### Hypervalent iodine catalysis

Effective hypervalent iodine(III)-catalyzed processes (for example, oxidative double C=C bond functionalization, oxidative cyclizations, CH-functionalization of carbonyl compounds, etc.) employing mainly peroxoacids or electric current as terminal oxidants were developed [139–145]. The key step in the catalytic cycle involving aryl iodides is the formation of iodine(III) species. Enantioselective oxidative processes mediated by chiral hypervalent iodine compounds were reviewed recently [146].

**Scheme 33:**
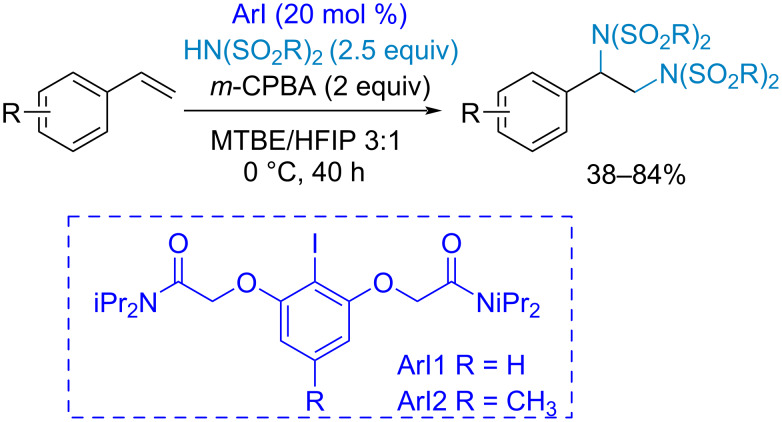
Iodoarene-organocatalyzed vinylarene diamination.

In the following example of styrene diamination by a chiral aryl iodide, the higher efficiency of the proposed catalyst compared to simpler aryl iodides was attributed to the additional stabilization of the I(III) intermediate by chelation via n–σ* interactions and hydrogen bonding [147] (Scheme 33).

**Scheme 34:**
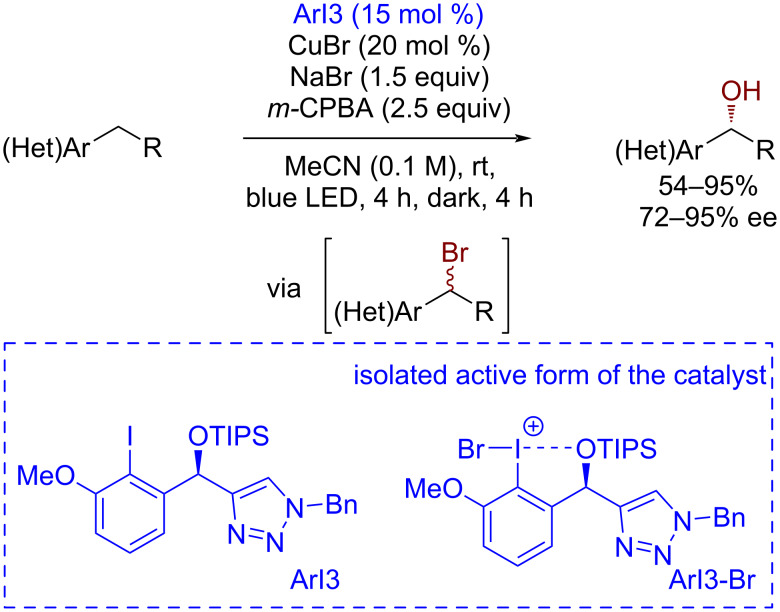
Iodoarene-organocatalyzed asymmetric CH-hydroxylation of benzylic substrates.

The enantioselective hydroxylation of benzylic positions was achieved using a chiral aryl iodide mediator [148] (Scheme 34). At the first stage, under the action of *m*-CPBA and sodium bromide an active form of the catalyst ArI3-Br is formed, in which the iodine–bromine bond is cleaved homolytically under visible light irradiation. The resulting iodoaryl cation radical abstracts the hydrogen atom from the benzylic position to form a benzyl radical, whose bromination gives the racemic benzyl bromide. The second step takes place without the participation of light and leads to only one enantiomer due to the assistance of the chiral copper complex.

**Scheme 35:**
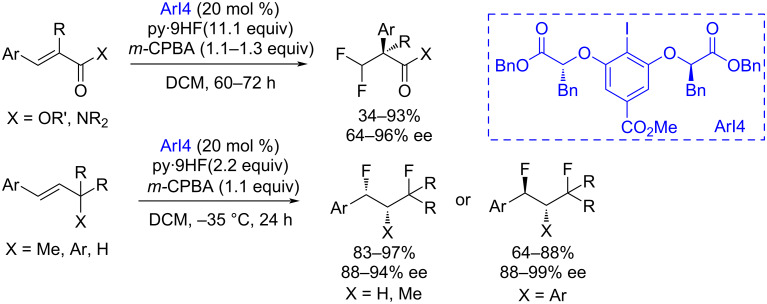
Iodoarene-organocatalyzed asymmetric difluorination of alkenes with migration of aryl or methyl groups.

An extraordinary example of an asymmetric difluorination of alkenes with the migration of aryl or methyl groups was shown using a chiral aryl iodide catalyst [149–150] (Scheme 35). Depending on the nature of the migrating group, two mechanisms are possible that determine the configuration of the final product.

**Scheme 36:**
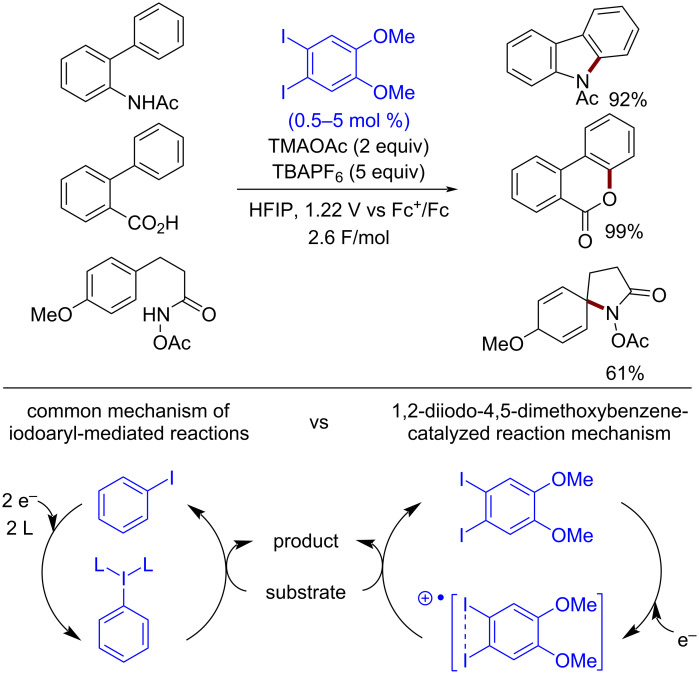
Examples of 1,2-diiodo-4,5-dimethoxybenzene-catalyzed electrochemical oxidative heterocyclizations.

A fundamentally different mechanism distinguishes the 1,2-diiodo-4,5-dimethoxybenzene catalyst from other aryl iodides. In contrast to the standard mechanism, in which iodine(III) is an active intermediate species, in the case of 1,2-diiodo-4,5-dimethoxybenzene, the iodine–iodine bonding interaction stabilizes the iodanyl radical intermediate (I(II) species, Scheme 36). This allows reactions to be carried out at lower potentials with lower catalyst loadings extending the applicability of the method to more labile substrates, such as those containing an anisole moiety [151] (Scheme 36).

One of the emerging areas in the chemistry of iodine(III) reagents with high synthetic potential is the visible-light induced I–O bond homolysis [152], which is currently employed mainly for the initiation of chain processes or stoichiometric oxidations, but definitely should find application in catalysis.

#### Examples of redox-active compounds of other classes as effective organocatalysts for oxidative transformations

**Scheme 37:**
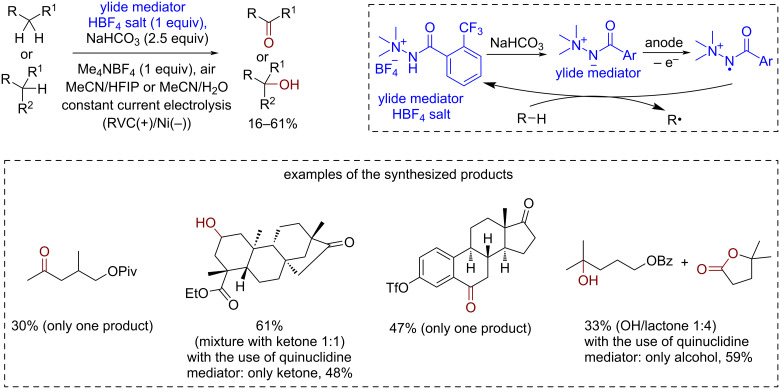
Electrochemical *N*-ammonium ylide-catalyzed CH-oxidation.

In addition to the widely used redox-organocatalyst classes discussed above, several novel structural types of redox-active molecules with rich potential for synthetic application emerged in the last years. For example, a new class of synthetically available and structurally tunable HAT mediators (*N*-ammonium ylides) was rationally designed for electrochemical CH-oxidation [153] (Scheme 37). By computational studies *N*-ammonium ylides were chosen for investigation due their high hydrogen binding energy and low deprotonation free energy. *N*-Ammonium ylides were used for the electrochemical oxidation of unactivated C–H bonds (Scheme 37). Ylides showed good selectivity and an unusual reactivity pattern in comparison with known mediators for CH-oxidation. For example, a methylene moiety oxidation mediated by *N*-ammonium ylides gave a ketone with significant quantities of a secondary alcohol in contrast to quinuclidine-mediated oxidation, which produced only the ketone. The oxidation of tertiary C–H bonds catalyzed by *N*-ammonium ylides occurred in low yields.

**Scheme 38:**
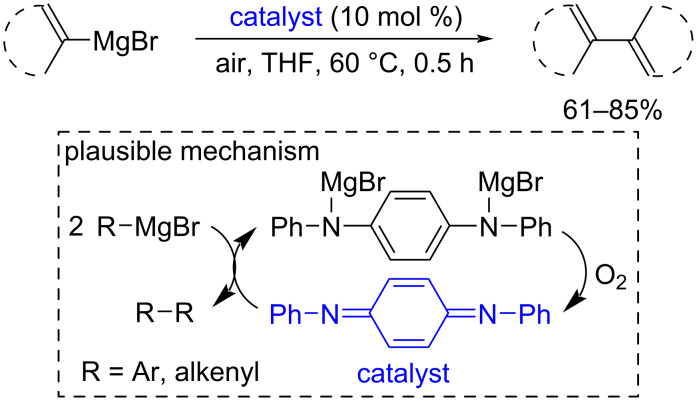
Oxidative dimerization of aryl- and alkenylmagnesium compounds catalyzed by quinonediimines.

Quinonediimines were used as redox-active organocatalysts for the oxidative coupling of aryl- and alkenylmagnesium compounds employing molecular oxygen as the terminal oxidant [154] (Scheme 38).

**Scheme 39:**
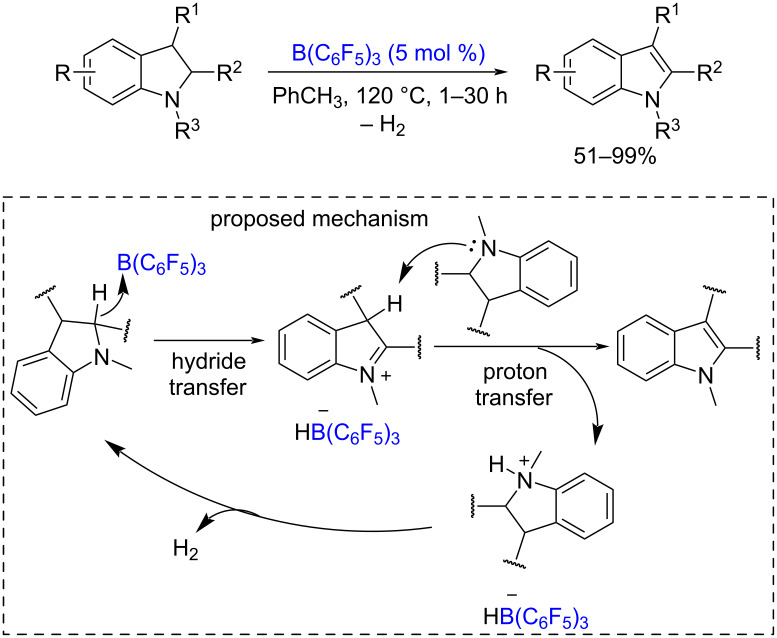
FLP-catalyzed dehydrogenation of N-substituted indolines.

Frustrated Lewis pairs (FLP) have gained much attention in the last decade due to unique reactivity, such as metal-free H_2_ activation for hydrogenation of various organic substrates. More recently, SET reactivity of FLP was discovered [155]. The FLP-catalyzed dehydrogenation of N-protected indolines with H_2_ release [156] is depicted in Scheme 39.

According to the proposed mechanism, the reaction starts with a hydride transfer from the indoline to B(C_6_F_5_)_3_. The resultant iminium ion is deprotonated by a second indoline molecule with the formation of an ammonium ion and the final indole. The ammonium ion reacts with a HB(C_6_F_5_)_3_ anion with the release of a H_2_ molecule and the regeneration of B(C_6_F_5_)_3_ for the next catalytic cycle.

## Conclusion

In conclusion, redox-active organic molecules represent an important class of organocatalysts for selective oxidative transformations. They are frequently used in combination with transition metal salts, photoredox catalysts, and in electrochemical oxidative processes. Currently, the most widely used redox-active compound classes for organocatalyzed oxidative processes are *N*-oxyl radicals, oxoammonium cations, amine cation radicals, thiyl radicals, quinones, dioxiranes and oxaziridines, and hypervalent iodine compounds. The main applications include selective CH-functionalization (hydroxylation, hydroperoxidation, halogenation, etc.), cross-dehydrogenative coupling and oxidative cyclization, alcohol oxidation, and the oxidation of other functional groups.

Compared to other types of organocatalysis (type I and type II in Scheme 1) reversible bonding and non-covalent interactions of redox-active molecules with a substrate are not very well studied and used. The selectivity in organocatalysis by redox-active molecules is controlled mainly by bond energies, redox-potentials, polar effects [157–158], and steric effects. Enantioselective transformations are relatively rare. Borrowing ideas from other types of organocatalysis for more precise selectivity control can be one of perspective research directions.

Another fundamental problem in the discussed redox-organocatalysis field is the understanding of the reactivity of catalytically active intermediates (usually radicals and cation radicals of limited stability) formed in situ. The study of connection between chemical structure and properties (stability to self-decay and various side processes, selectivity depending on reaction conditions) is the key to the future rational design of improved, robust, and selective redox-organocatalysts.
